# Diammonium tetra­kis­(iso­thio­cyanato)­zincate–1,4,10,13,16-hexa­oxa­cyclo­octa­deca­ne–water (1/2/1)

**DOI:** 10.1107/S1600536813019739

**Published:** 2013-07-24

**Authors:** K. Showrilu, K. Rajarajan, M. NizamMohideen

**Affiliations:** a Research and Development Centre, Bharathiyar University, Coimbatore 641 046, India; b Department of Physics, Rajeswari Vedachalam Government Arts College, Chengalpet 603 001, India; c Department of Physics, The New College (Autonomous), Chennai 600 014, India

## Abstract

The title compound, (NH_4_)_2_[Zn(NCS)_4_]·2C_12_H_24_O_6_·H_2_O, the result of the reaction of ammonium thio­cyanate, 18-crown-6 and zinc(II) chloride in aqueous solution, exhibits an unusual supra­molecular structure. The Zn atom, two of the thio­cyanate chains and a water mol­ecule, disordered over two positions, lie on a mirror plane. The macrocycle adopts a conformation with approximate *D*_3*d*_ symmetry. The ammonium mol­ecules are contained within the bowl of the macrocycle *via* extensive N—H⋯O hydrogen bonds and the complex mol­ecules are linked *via* N—H⋯S hydrogen bonds, forming chains along the *c*-axis direction. The macrocycle is disordered over two positions [refined occupancy ratio = 0.666 (8):0.334 (8)]. The S atoms of two iso­thio­cyanate ligands are disordered within and about the mirror plane.

## Related literature

For background to crown ether/ammonium ion complexes, see: Fender *et al.* (2002 ▶); Kryatova *et al.* (2004 ▶); Akutagawa *et al.* (2002 ▶); Ramesh *et al.* (2012 ▶).
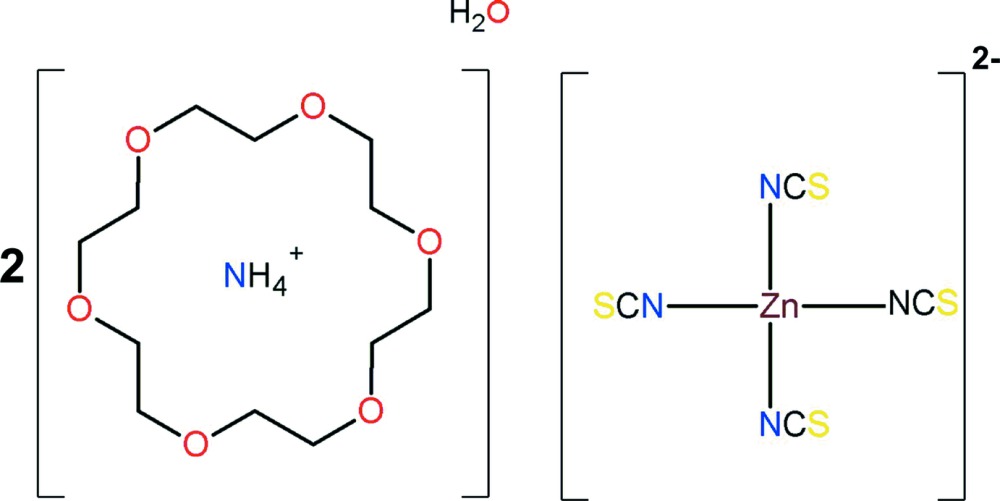


## Experimental

### 

#### Crystal data

(NH_4_)_2_[Zn(NCS)_4_]·2C_12_H_24_O_6_·H_2_O
*M* *_r_* = 880.41
Orthorhombic, 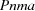

*a* = 22.7875 (12) Å
*b* = 23.6254 (12) Å
*c* = 8.5593 (5) Å
*V* = 4608.0 (4) Å^3^
*Z* = 4
Mo *K*α radiation
μ = 0.77 mm^−1^
*T* = 293 K
0.30 × 0.25 × 0.20 mm

#### Data collection

Bruker Kappa APEXII CCD diffractometer
Absorption correction: multi-scan (*SADABS*; Sheldrick, 2004 ▶) *T*_min_ = 0.802, *T*_max_ = 0.861
24101 measured reflections
4641 independent reflections
2467 reflections with *I* > 2σ(*I*)
*R*_int_ = 0.058

#### Refinement

*R*[*F* ^2^ > 2σ(*F* ^2^)] = 0.052
*wR*(*F* ^2^) = 0.175
*S* = 1.00
4641 reflections
454 parameters
276 restraints
H atoms treated by a mixture of independent and constrained refinement
Δρ_max_ = 0.72 e Å^−3^
Δρ_min_ = −0.24 e Å^−3^

### 

Data collection: *APEX2* (Bruker, 2004 ▶); cell refinement: *APEX2* and *SAINT* (Bruker, 2004 ▶); data reduction: *SAINT* and *XPREP* (Bruker, 2004 ▶); program(s) used to solve structure: *SHELXS97* (Sheldrick, 2008 ▶); program(s) used to refine structure: *SHELXL97* (Sheldrick, 2008 ▶); molecular graphics: *ORTEP-3 for Windows* (Farrugia, 2012 ▶) and *Mercury* (Macrae *et al.*, 2008 ▶); software used to prepare material for publication: *WinGX* (Farrugia, 2012 ▶) and *PLATON* (Spek, 2009 ▶).

## Supplementary Material

Crystal structure: contains datablock(s) global, I. DOI: 10.1107/S1600536813019739/su2617sup1.cif

Structure factors: contains datablock(s) I. DOI: 10.1107/S1600536813019739/su2617Isup2.hkl

Additional supplementary materials:  crystallographic information; 3D view; checkCIF report

## Figures and Tables

**Table 1:** Hydrogen-bond geometry (Å, °)

*D*—H⋯*A*	*D*—H	H⋯*A*	*D*⋯*A*	*D*—H⋯*A*
N4—H4*H*⋯O1	0.88 (1)	2.44 (4)	3.069 (8)	129 (4)
N4—H4*E*⋯O2	0.87 (1)	2.06 (1)	2.934 (8)	176 (5)
N4—H4*G*⋯O4	0.88 (1)	1.97 (2)	2.829 (7)	166 (4)
N4—H4*G*⋯O5	0.88 (1)	2.53 (4)	3.010 (8)	115 (3)
N4—H4*H*⋯O6	0.88 (1)	2.05 (3)	2.850 (8)	150 (5)
N4—H4*F*⋯S1^i^	0.87 (1)	2.63 (2)	3.441 (4)	156 (4)

Symmetry code: (i) 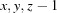.

**Fig. 1.**
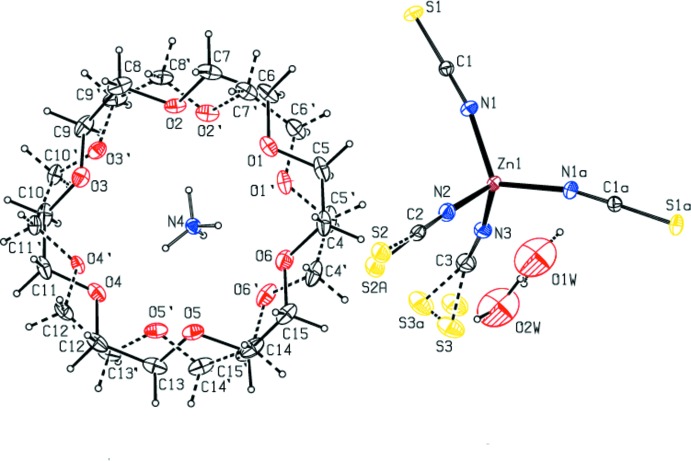
Crystal structure of the title compound with atom labelling. Displacement ellipsoids are drawn at the 30% probability level [symmetry code: (a) x, -y+1/2, z; the disordered fraction is shown with dashed bonds and atom labels with prefix '].

**Fig. 2.**
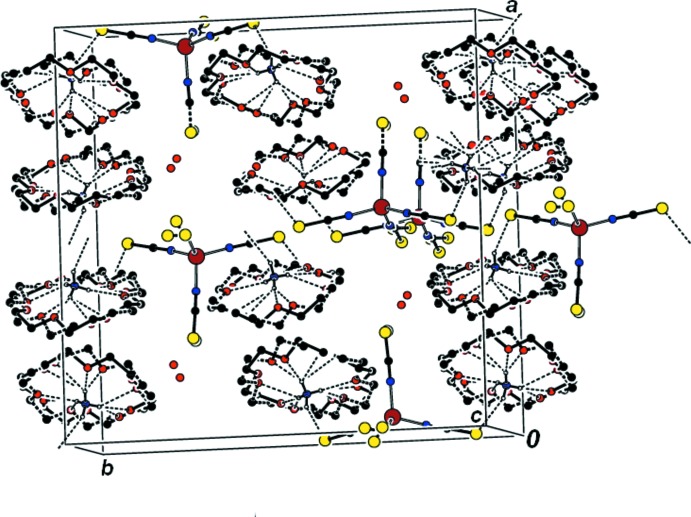
A view along the c axis of the crystal packing of the title compound. Hydrogen bonds are shown as dashed lines (see Table 1 for details).

## supplementary crystallographic information

### Comment

There is currently significant interest in crown ethers because of their ability to form non-covalent, hydrogen bonding complexes with ammonium cations both in the solid state and in solution (Fender *et al.*, 2002; Kryatova *et al.*, 2004; Akutagawa *et al.*, 2002). Recently, the crystal structure of *catena*-Poly[ammonium(cadmium-tri-lthiocyanato κ^4^*S*:*N*; κ^2^*N*:*S*) -1,4,10,13,16- hexaoxacyclooctadecane (1/1)] (I), obtained in our laboratory, has been reported (Ramesh *et al.*, 2012). In continuation of our studies of compounds containing 18-crown-6 macrocycles and ammonium cations NH_4_^+^, we describe herein the crystal structure of the title compound (II), which is isostructural with (I).

The reaction of ammonium thiocyanate, 18-crown-6 and Zinc (II) chloride in aqueous solution yields the title compound, Fig. 1. All bond lengths and angles are normal and correspond to those reported for (I) (Ramesh *et al.*, 2012). The C—S [average value of 1.658 (2) Å] and C—N [average value of 1.116 (2) Å] bond lengths indicate the presence of double-bond character. The zinc atom, Zn1, two of the thiocyanate chains [N2—C2—S2 and N3—C3—S3; symmetry code: *x*, -*y* + 1/2, *z*] and the water molecule lie in a mirror plane, except one of the disordered component sulfur atoms, which is inclined at quite an angle to the *ac* plane. The thiocyanate (N1—C1—S1 = 178.2 (4) °) ligands are almost linear.

The macrocycle adopts a conformation with approximate D_3d_ symmetry, with all O—C—C—O torsion angles being *gauche* and alternating in sign, and all C—O—C—C torsion angles being *anti*.

The sulfur atoms (S2 and S3) of the thiocyanate chains are disordered with large displacement parameters for the S atoms and short C—S bond lengths. The disorder over two positions was modelled and the site occupancies refined to 0.39 (9) and 0.61 (9) for atom S2 and 0.376 (9) and 0.248 (18) for atom S3. The entire crown either molecule is disordered, as detectable from the large displacement parameters for C and O atoms and short C—C and C—O bond lengths. The disorder over two positions was modelled and the site occupancies refined to 0.666 (9) and 0.334 (9) for carbon and oxygen atoms. The water molecule is disordered, as detectable from the large displacement parameters for the O atoms. The disorder over two positions was modelled and the site occupancies refined to 0.425 (15) and 0.575 (15). for carbon and oxygen atoms. The geometry was regularized by soft restraints.

The ammonium cations are contained within the bowl of the macrocycle *via* extensive N—H···O hydrogen bonding. The N—H···O [2.830 (7) to 3.074 (7) Å] and N—H···S [3.442 (4) Å] hydrogen bond lengths are within the usual range (Table 1 and Fig. 2).

In the crystal, the complex molecules are linked *via* N—H···S hydrogen bonds forming chains along the *c* axis (Table 1 and Fig. 2).

### Experimental

A mixture of 18-crown-6, ammonium thiocyanate and Zinc (II) chloride were dissolved in an aqueous solution in the molar ratio 2:4:1 and thoroughly mixed for two hours to obtain a homogeneous mixture. The solution was allowed to evaporate slowly at ambient temperature. Colourless single crystals suitable for single-crystal X-ray diffraction analysis were obtained in a week.

### Refinement

The sulfur atoms (S2 and S3) of the thiocyanate chain are disordered over two positions with refined occupancies of 0.39 (9) and 0.61 (9) for atom S2, and 0.376 (9) and 0.248 (18) for atom S3. The entire crown either molecule is disordered over two positions with refined occupancies of 0.666 (9) and 0.334 (9). The water molecule is disordered over two positions with refined occupancies of 0.425 (15) and 0.575 (15). The corresponding bond distances involving the disordered atoms were restrained to be equal.

The N-bound H (N—H = 0.87 Å) atoms was located in difference map and refined in the riding mode approximation. C-bound H-atoms were placed in calculated positions [C—H 0.97 Å, *U*_iso_(H) 1.2*U*_eq_(C)] and were included in the refinement in the riding model approximation. The water H-atom, whose O atom lies on a mirror plane, was similar treated [O—H 0.61–0.90 Å].

### Crystal data

**Table:**

(NH_4_)_2_[Zn(NCS)_4_]·2C_12_H_24_O_6_·H_2_O	*F*(000) = 1864
*M**_r_* = 880.41	*D*_x_ = 1.269 Mg m^−^^3^
Orthorhombic, *P**n**m**a*	Mo *K*α radiation, λ = 0.71073 Å
Hall symbol: -P 2ac 2n	Cell parameters from 5119 reflections
*a* = 22.7875 (12) Å	θ = 2.6–26.7°
*b* = 23.6254 (12) Å	µ = 0.77 mm^−^^1^
*c* = 8.5593 (5) Å	*T* = 293 K
*V* = 4608.0 (4) Å^3^	Block, colourless
*Z* = 4	0.30 × 0.25 × 0.20 mm

### Data collection

**Table:**

Bruker Kappa APEXII CCD diffractometer	4641 independent reflections
Radiation source: fine-focus sealed tube	2467 reflections with *I* > 2σ(*I*)
Graphite monochromator	*R*_int_ = 0.058
ω and φ scan	θ_max_ = 26.0°, θ_min_ = 2.5°
Absorption correction: multi-scan (*SADABS*; Sheldrick, 2004)	*h* = −24→28
*T*_min_ = 0.802, *T*_max_ = 0.861	*k* = −19→29
24101 measured reflections	*l* = −10→9

### Refinement

**Table:**

Refinement on *F*^2^	Primary atom site location: structure-invariant direct methods
Least-squares matrix: full	Secondary atom site location: difference Fourier map
*R*[*F*^2^ > 2σ(*F*^2^)] = 0.052	Hydrogen site location: inferred from neighbouring sites
*wR*(*F*^2^) = 0.175	H atoms treated by a mixture of independent and constrained refinement
*S* = 1.00	*w* = 1/[σ^2^(*F*_o_^2^) + (0.0932*P*)^2^ + 0.2607*P*] where *P* = (*F*_o_^2^ + 2*F*_c_^2^)/3
4641 reflections	(Δ/σ)_max_ = 0.001
454 parameters	Δρ_max_ = 0.72 e Å^−^^3^
276 restraints	Δρ_min_ = −0.24 e Å^−^^3^

### Special details

**Table:**

Geometry. All e.s.d.'s (except the e.s.d. in the dihedral angle between two l.s. planes) are estimated using the full covariance matrix. The cell e.s.d.'s are taken into account individually in the estimation of e.s.d.'s in distances, angles and torsion angles; correlations between e.s.d.'s in cell parameters are only used when they are defined by crystal symmetry. An approximate (isotropic) treatment of cell e.s.d.'s is used for estimating e.s.d.'s involving l.s. planes.
Refinement. Refinement of *F*^2^ against ALL reflections. The weighted *R*-factor *wR* and goodness of fit *S* are based on *F*^2^, conventional *R*-factors *R* are based on *F*, with *F* set to zero for negative *F*^2^. The threshold expression of *F*^2^ > σ(*F*^2^) is used only for calculating *R*-factors(gt) *etc*. and is not relevant to the choice of reflections for refinement. *R*-factors based on *F*^2^ are statistically about twice as large as those based on *F*, and *R*- factors based on ALL data will be even larger.

### Fractional atomic coordinates and isotropic or equivalent isotropic displacement parameters (Å^2^)

**Table:**

	*x*	*y*	*z*	*U*_iso_*/*U*_eq_	Occ. (<1)
C1	0.51074 (15)	0.13492 (16)	0.9699 (5)	0.0534 (10)	
C2	0.6728 (3)	0.2500	0.7518 (7)	0.0668 (16)	
C3	0.4978 (4)	0.2500	0.4932 (11)	0.094 (2)	
O1	0.6093 (3)	0.0412 (3)	0.5640 (8)	0.086 (2)	0.666 (8)
O2	0.6025 (4)	−0.0710 (3)	0.4650 (8)	0.082 (2)	0.666 (8)
O3	0.6681 (3)	−0.1007 (3)	0.1940 (9)	0.079 (2)	0.666 (8)
O4	0.6950 (3)	−0.0163 (3)	−0.0249 (8)	0.0798 (19)	0.666 (8)
O5	0.6998 (3)	0.0974 (4)	0.0798 (7)	0.083 (2)	0.666 (8)
O6	0.6318 (3)	0.1232 (3)	0.3415 (9)	0.092 (2)	0.666 (8)
C4	0.6209 (4)	0.1375 (4)	0.4993 (11)	0.103 (3)	0.666 (8)
H4A	0.6025	0.1744	0.5053	0.124*	0.666 (8)
H4B	0.6576	0.1388	0.5567	0.124*	0.666 (8)
C5	0.5811 (4)	0.0935 (4)	0.5696 (12)	0.106 (3)	0.666 (8)
H5A	0.5721	0.1034	0.6770	0.127*	0.666 (8)
H5B	0.5446	0.0918	0.5115	0.127*	0.666 (8)
C6	0.5761 (5)	0.0013 (5)	0.6449 (14)	0.097 (4)	0.666 (8)
H6A	0.5363	0.0007	0.6046	0.116*	0.666 (8)
H6B	0.5745	0.0110	0.7549	0.116*	0.666 (8)
C7	0.6040 (4)	−0.0556 (5)	0.6243 (9)	0.102 (3)	0.666 (8)
H7A	0.6443	−0.0544	0.6603	0.122*	0.666 (8)
H7B	0.5831	−0.0835	0.6859	0.122*	0.666 (8)
C8	0.6256 (5)	−0.1260 (4)	0.4391 (12)	0.103 (3)	0.666 (8)
H8A	0.6009	−0.1539	0.4898	0.123*	0.666 (8)
H8B	0.6646	−0.1286	0.4840	0.123*	0.666 (8)
C9	0.6284 (4)	−0.1378 (4)	0.2670 (12)	0.094 (3)	0.666 (8)
H9A	0.6407	−0.1766	0.2500	0.113*	0.666 (8)
H9B	0.5897	−0.1331	0.2214	0.113*	0.666 (8)
C10	0.6751 (5)	−0.1127 (5)	0.0337 (12)	0.093 (4)	0.666 (8)
H10A	0.6377	−0.1093	−0.0199	0.111*	0.666 (8)
H10B	0.6895	−0.1510	0.0198	0.111*	0.666 (8)
C11	0.7185 (4)	−0.0709 (4)	−0.0321 (10)	0.091 (3)	0.666 (8)
H11A	0.7547	−0.0724	0.0273	0.109*	0.666 (8)
H11B	0.7273	−0.0805	−0.1397	0.109*	0.666 (8)
C12	0.7343 (5)	0.0238 (5)	−0.0886 (15)	0.094 (4)	0.666 (8)
H12A	0.7426	0.0148	−0.1969	0.113*	0.666 (8)
H12B	0.7709	0.0237	−0.0308	0.113*	0.666 (8)
C13	0.7052 (4)	0.0806 (5)	−0.0767 (10)	0.098 (3)	0.666 (8)
H13A	0.7282	0.1084	−0.1333	0.118*	0.666 (8)
H13B	0.6666	0.0788	−0.1241	0.118*	0.666 (8)
C14	0.6687 (5)	0.1489 (5)	0.0924 (14)	0.107 (4)	0.666 (8)
H14A	0.6296	0.1447	0.0488	0.129*	0.666 (8)
H14B	0.6889	0.1785	0.0351	0.129*	0.666 (8)
C15	0.6647 (5)	0.1645 (4)	0.2617 (13)	0.107 (3)	0.666 (8)
H15A	0.7038	0.1669	0.3062	0.128*	0.666 (8)
H15B	0.6460	0.2012	0.2726	0.128*	0.666 (8)
O1'	0.6101 (6)	0.0748 (7)	0.5055 (18)	0.095 (4)	0.334 (8)
O2'	0.5880 (8)	−0.0418 (7)	0.489 (2)	0.094 (4)	0.334 (8)
O3'	0.6481 (6)	−0.1061 (6)	0.2641 (16)	0.073 (4)	0.334 (8)
O4'	0.6913 (5)	−0.0500 (6)	0.0013 (14)	0.062 (3)	0.334 (8)
O5'	0.7128 (6)	0.0659 (6)	0.0284 (19)	0.082 (4)	0.334 (8)
O6'	0.6437 (7)	0.1284 (6)	0.2342 (19)	0.103 (5)	0.334 (8)
C4'	0.6350 (10)	0.1591 (9)	0.373 (2)	0.115 (6)	0.334 (8)
H4'1	0.6223	0.1974	0.3495	0.138*	0.334 (8)
H4'2	0.6714	0.1613	0.4319	0.138*	0.334 (8)
C5'	0.5891 (10)	0.1293 (8)	0.468 (3)	0.118 (7)	0.334 (8)
H5'1	0.5809	0.1504	0.5623	0.142*	0.334 (8)
H5'2	0.5531	0.1263	0.4079	0.142*	0.334 (8)
C6'	0.5720 (9)	0.0449 (9)	0.604 (2)	0.103 (6)	0.334 (8)
H6'1	0.5334	0.0432	0.5563	0.124*	0.334 (8)
H6'2	0.5683	0.0649	0.7024	0.124*	0.334 (8)
C7'	0.5935 (12)	−0.0141 (10)	0.634 (2)	0.098 (7)	0.334 (8)
H7'1	0.6340	−0.0137	0.6686	0.118*	0.334 (8)
H7'2	0.5697	−0.0326	0.7132	0.118*	0.334 (8)
C8'	0.6053 (10)	−0.0980 (7)	0.513 (2)	0.094 (6)	0.334 (8)
H8'1	0.5787	−0.1159	0.5865	0.113*	0.334 (8)
H8'2	0.6444	−0.0987	0.5586	0.113*	0.334 (8)
C9'	0.6055 (9)	−0.1305 (8)	0.363 (2)	0.093 (6)	0.334 (8)
H9'1	0.6148	−0.1699	0.3822	0.112*	0.334 (8)
H9'2	0.5671	−0.1285	0.3139	0.112*	0.334 (8)
C10'	0.6499 (6)	−0.1330 (6)	0.1179 (19)	0.071 (4)	0.334 (8)
H10C	0.6134	−0.1267	0.0621	0.085*	0.334 (8)
H10D	0.6553	−0.1735	0.1312	0.085*	0.334 (8)
C11'	0.7003 (6)	−0.1085 (6)	0.028 (3)	0.058 (4)	0.334 (8)
H11C	0.7365	−0.1139	0.0858	0.070*	0.334 (8)
H11D	0.7041	−0.1279	−0.0716	0.070*	0.334 (8)
C12'	0.7390 (6)	−0.0224 (7)	−0.0687 (18)	0.080 (4)	0.334 (8)
H12C	0.7514	−0.0431	−0.1608	0.096*	0.334 (8)
H12D	0.7717	−0.0209	0.0038	0.096*	0.334 (8)
C13'	0.7211 (12)	0.0367 (9)	−0.114 (2)	0.082 (6)	0.334 (8)
H13C	0.7515	0.0547	−0.1755	0.098*	0.334 (8)
H13D	0.6850	0.0362	−0.1738	0.098*	0.334 (8)
C14'	0.6902 (10)	0.1195 (6)	−0.007 (3)	0.105 (6)	0.334 (8)
H14C	0.6521	0.1152	−0.0563	0.125*	0.334 (8)
H14D	0.7160	0.1382	−0.0809	0.125*	0.334 (8)
C15'	0.6838 (11)	0.1560 (10)	0.135 (3)	0.105 (7)	0.334 (8)
H15C	0.7213	0.1605	0.1871	0.127*	0.334 (8)
H15D	0.6692	0.1932	0.1067	0.127*	0.334 (8)
N1	0.52011 (15)	0.18067 (14)	0.9297 (4)	0.0703 (10)	
N2	0.6247 (3)	0.2500	0.7865 (7)	0.0768 (16)	
N3	0.5010 (3)	0.2500	0.6166 (7)	0.0796 (16)	
N4	0.61488 (17)	0.01292 (18)	0.2144 (5)	0.0594 (9)	
S1	0.49673 (5)	0.07035 (4)	1.02060 (15)	0.0704 (4)	
S2	0.7443 (4)	0.2500	0.740 (7)	0.100 (6)	0.39 (9)
S2A	0.7397 (6)	0.2500	0.684 (5)	0.098 (5)	0.61 (9)
S3	0.5017 (4)	0.2703 (4)	0.3036 (5)	0.138 (3)	0.376 (9)
S3A	0.4660 (14)	0.2500	0.3119 (17)	0.161 (7)	0.248 (18)
Zn1	0.54016 (3)	0.2500	0.82110 (8)	0.0587 (3)	
O1W	0.3179 (18)	0.2500	0.361 (5)	0.328 (15)	0.425 (15)
O2W	0.3443 (14)	0.2500	0.199 (3)	0.328 (15)	0.575 (15)
H1W	0.31 (2)	0.2500	0.258 (11)	0.492*	0.425 (15)
H2W	0.289 (15)	0.2500	0.43 (4)	0.492*	0.425 (15)
H3W	0.327 (15)	0.2500	0.29 (2)	0.492*	0.575 (15)
H4W	0.384 (3)	0.2500	0.19 (5)	0.492*	0.575 (15)
H4E	0.609 (2)	−0.0122 (17)	0.288 (4)	0.11 (2)*	
H4F	0.5826 (12)	0.016 (2)	0.160 (5)	0.099 (18)*	
H4G	0.6438 (14)	0.0081 (19)	0.148 (4)	0.097 (17)*	
H4H	0.624 (2)	0.0393 (16)	0.282 (5)	0.11 (2)*	

### Atomic displacement parameters (Å^2^)

**Table:**

	*U*^11^	*U*^22^	*U*^33^	*U*^12^	*U*^13^	*U*^23^
C1	0.048 (2)	0.053 (2)	0.059 (3)	0.0033 (18)	0.0027 (18)	0.004 (2)
C2	0.089 (5)	0.043 (3)	0.069 (4)	0.000	0.003 (4)	0.000
C3	0.110 (6)	0.090 (5)	0.083 (6)	0.000	−0.001 (5)	0.000
O1	0.080 (4)	0.112 (5)	0.067 (4)	0.007 (4)	0.016 (3)	−0.017 (4)
O2	0.097 (5)	0.083 (5)	0.065 (4)	−0.013 (4)	−0.009 (3)	0.026 (4)
O3	0.085 (5)	0.073 (4)	0.081 (5)	0.002 (3)	−0.018 (4)	−0.011 (4)
O4	0.061 (4)	0.113 (5)	0.065 (4)	0.007 (4)	0.013 (3)	−0.003 (4)
O5	0.082 (4)	0.087 (5)	0.080 (5)	−0.003 (4)	−0.014 (3)	0.031 (4)
O6	0.110 (4)	0.070 (4)	0.097 (6)	0.012 (3)	−0.008 (4)	−0.008 (4)
C4	0.108 (7)	0.098 (7)	0.102 (7)	0.038 (6)	−0.010 (6)	−0.047 (6)
C5	0.094 (6)	0.137 (8)	0.086 (7)	0.031 (7)	0.011 (5)	−0.042 (7)
C6	0.096 (8)	0.139 (10)	0.056 (6)	−0.014 (7)	0.023 (5)	−0.013 (6)
C7	0.114 (6)	0.133 (8)	0.059 (6)	−0.031 (6)	0.004 (5)	0.023 (6)
C8	0.120 (8)	0.084 (6)	0.105 (8)	−0.019 (6)	−0.026 (6)	0.030 (6)
C9	0.105 (7)	0.057 (5)	0.119 (9)	0.002 (5)	−0.032 (6)	−0.006 (6)
C10	0.092 (9)	0.089 (7)	0.097 (7)	0.030 (6)	−0.022 (7)	−0.033 (6)
C11	0.078 (6)	0.131 (9)	0.063 (5)	0.038 (7)	0.002 (5)	−0.034 (6)
C12	0.061 (6)	0.155 (10)	0.068 (7)	−0.007 (6)	0.017 (5)	0.000 (8)
C13	0.085 (6)	0.138 (9)	0.072 (6)	−0.023 (6)	0.007 (5)	0.022 (7)
C14	0.122 (7)	0.075 (7)	0.124 (9)	−0.010 (6)	−0.040 (7)	0.036 (7)
C15	0.129 (7)	0.058 (5)	0.133 (9)	0.009 (5)	−0.017 (7)	0.012 (6)
O1'	0.087 (7)	0.101 (10)	0.097 (10)	0.023 (8)	0.017 (7)	−0.039 (8)
O2'	0.098 (9)	0.108 (11)	0.075 (8)	−0.022 (8)	−0.004 (7)	0.019 (8)
O3'	0.072 (8)	0.053 (6)	0.094 (10)	−0.012 (6)	−0.007 (6)	0.004 (7)
O4'	0.047 (6)	0.076 (8)	0.063 (7)	0.009 (6)	0.018 (5)	−0.014 (7)
O5'	0.086 (8)	0.095 (9)	0.066 (8)	−0.023 (7)	−0.022 (7)	0.027 (8)
O6'	0.123 (10)	0.056 (7)	0.129 (14)	0.007 (6)	−0.046 (9)	−0.009 (8)
C4'	0.141 (13)	0.061 (10)	0.143 (16)	0.026 (10)	−0.024 (12)	−0.034 (11)
C5'	0.112 (12)	0.095 (11)	0.147 (15)	0.034 (11)	−0.015 (12)	−0.051 (11)
C6'	0.106 (12)	0.123 (14)	0.081 (11)	−0.001 (12)	0.026 (10)	−0.014 (11)
C7'	0.102 (13)	0.123 (14)	0.070 (12)	−0.014 (12)	0.020 (10)	0.002 (12)
C8'	0.104 (11)	0.094 (12)	0.084 (12)	−0.036 (10)	0.006 (10)	0.031 (10)
C9'	0.113 (12)	0.075 (10)	0.091 (13)	−0.040 (9)	−0.017 (10)	0.024 (10)
C10'	0.063 (8)	0.051 (7)	0.099 (11)	0.007 (6)	−0.011 (8)	−0.016 (8)
C11'	0.044 (9)	0.058 (8)	0.074 (9)	0.003 (7)	−0.007 (7)	−0.024 (7)
C12'	0.087 (10)	0.102 (11)	0.052 (9)	−0.019 (9)	0.017 (7)	−0.023 (9)
C13'	0.083 (12)	0.114 (13)	0.049 (10)	−0.023 (11)	0.004 (9)	0.022 (9)
C14'	0.124 (12)	0.084 (11)	0.106 (13)	−0.024 (10)	−0.035 (11)	0.035 (11)
C15'	0.140 (13)	0.057 (9)	0.120 (16)	−0.020 (10)	−0.046 (12)	0.011 (10)
N1	0.087 (2)	0.0477 (19)	0.076 (3)	−0.0038 (17)	0.0057 (19)	0.0091 (19)
N2	0.069 (3)	0.063 (3)	0.099 (5)	0.000	0.015 (3)	0.000
N3	0.110 (5)	0.068 (3)	0.060 (4)	0.000	0.009 (4)	0.000
N4	0.061 (3)	0.067 (2)	0.051 (3)	0.007 (2)	−0.001 (2)	−0.002 (2)
S1	0.0688 (7)	0.0521 (6)	0.0903 (9)	−0.0072 (5)	−0.0075 (6)	0.0253 (6)
S2	0.080 (5)	0.099 (5)	0.121 (17)	0.000	0.029 (5)	0.000
S2A	0.070 (3)	0.104 (3)	0.121 (12)	0.000	0.025 (4)	0.000
S3	0.149 (6)	0.198 (9)	0.067 (3)	−0.007 (4)	−0.005 (3)	0.016 (3)
S3A	0.234 (17)	0.175 (14)	0.076 (7)	0.000	0.019 (11)	0.000
Zn1	0.0725 (5)	0.0362 (3)	0.0674 (5)	0.000	0.0085 (4)	0.000
O1W	0.39 (3)	0.34 (2)	0.25 (3)	0.000	−0.20 (3)	0.000
O2W	0.39 (3)	0.34 (2)	0.25 (3)	0.000	−0.20 (3)	0.000

### Geometric parameters (Å, º)

**Table:**

C1—N1	1.154 (4)	O2'—C7'	1.410 (10)
C1—S1	1.618 (4)	O3'—C10'	1.405 (10)
C2—N2	1.136 (7)	O3'—C9'	1.410 (10)
C2—S2A	1.631 (9)	O4'—C12'	1.403 (9)
C2—S2	1.632 (10)	O4'—C11'	1.414 (10)
C3—N3	1.058 (8)	O5'—C14'	1.403 (10)
C3—S3^i^	1.695 (10)	O5'—C13'	1.409 (10)
C3—S3	1.695 (10)	O6'—C15'	1.406 (10)
C3—S3A	1.714 (12)	O6'—C4'	1.409 (10)
O1—C6	1.393 (9)	C4'—C5'	1.497 (10)
O1—C5	1.394 (8)	C4'—H4'1	0.9700
O2—C7	1.411 (8)	C4'—H4'2	0.9700
O2—C8	1.420 (8)	C5'—H5'1	0.9700
O3—C9	1.406 (8)	C5'—H5'2	0.9700
O3—C10	1.410 (8)	C6'—C7'	1.501 (10)
O4—C11	1.398 (7)	C6'—H6'1	0.9700
O4—C12	1.411 (9)	C6'—H6'2	0.9700
O5—C13	1.402 (8)	C7'—H7'1	0.9700
O5—C14	1.413 (9)	C7'—H7'2	0.9700
O6—C15	1.407 (8)	C8'—C9'	1.500 (10)
O6—C4	1.414 (8)	C8'—H8'1	0.9700
C4—C5	1.504 (8)	C8'—H8'2	0.9700
C4—H4A	0.9700	C9'—H9'1	0.9700
C4—H4B	0.9700	C9'—H9'2	0.9700
C5—H5A	0.9700	C10'—C11'	1.500 (10)
C5—H5B	0.9700	C10'—H10C	0.9700
C6—C7	1.499 (9)	C10'—H10D	0.9700
C6—H6A	0.9700	C11'—H11C	0.9700
C6—H6B	0.9700	C11'—H11D	0.9700
C7—H7A	0.9700	C12'—C13'	1.506 (10)
C7—H7B	0.9700	C12'—H12C	0.9700
C8—C9	1.501 (8)	C12'—H12D	0.9700
C8—H8A	0.9700	C13'—H13C	0.9700
C8—H8B	0.9700	C13'—H13D	0.9700
C9—H9A	0.9700	C14'—C15'	1.499 (10)
C9—H9B	0.9700	C14'—H14C	0.9700
C10—C11	1.507 (8)	C14'—H14D	0.9700
C10—H10A	0.9700	C15'—H15C	0.9700
C10—H10B	0.9700	C15'—H15D	0.9700
C11—H11A	0.9700	N1—Zn1	1.938 (3)
C11—H11B	0.9700	N2—Zn1	1.948 (6)
C12—C13	1.501 (8)	N3—Zn1	1.964 (7)
C12—H12A	0.9700	N4—H4E	0.873 (10)
C12—H12B	0.9700	N4—H4F	0.874 (10)
C13—H13A	0.9700	N4—H4G	0.878 (10)
C13—H13B	0.9700	N4—H4H	0.878 (10)
C14—C15	1.497 (9)	S3—S3^i^	0.957 (17)
C14—H14A	0.9700	Zn1—N1^i^	1.938 (3)
C14—H14B	0.9700	O1W—H1W	0.900 (11)
C15—H15A	0.9700	O1W—H2W	0.899 (10)
C15—H15B	0.9700	O1W—H3W	0.61 (7)
O1'—C6'	1.401 (10)	O2W—H1W	0.9 (5)
O1'—C5'	1.411 (10)	O2W—H3W	0.900 (10)
O2'—C8'	1.401 (10)	O2W—H4W	0.898 (11)
			
N1—C1—S1	178.1 (4)	O6'—C4'—H4'1	110.1
N2—C2—S2A	174.3 (16)	C5'—C4'—H4'1	110.1
N2—C2—S2	168 (2)	O6'—C4'—H4'2	110.1
N3—C3—S3^i^	162.2 (5)	C5'—C4'—H4'2	110.1
N3—C3—S3	162.2 (5)	H4'1—C4'—H4'2	108.4
N3—C3—S3A	158.9 (13)	O1'—C5'—C4'	108.5 (17)
C6—O1—C5	109.4 (8)	O1'—C5'—H5'1	110.0
C7—O2—C8	112.2 (8)	C4'—C5'—H5'1	110.0
C9—O3—C10	112.3 (7)	O1'—C5'—H5'2	110.0
C11—O4—C12	111.0 (8)	C4'—C5'—H5'2	110.0
C13—O5—C14	111.2 (9)	H5'1—C5'—H5'2	108.4
C15—O6—C4	113.1 (9)	O1'—C6'—C7'	112 (2)
O6—C4—C5	108.9 (7)	O1'—C6'—H6'1	109.3
O6—C4—H4A	109.9	C7'—C6'—H6'1	109.3
C5—C4—H4A	109.9	O1'—C6'—H6'2	109.3
O6—C4—H4B	109.9	C7'—C6'—H6'2	109.3
C5—C4—H4B	109.9	H6'1—C6'—H6'2	108.0
H4A—C4—H4B	108.3	O2'—C7'—C6'	104.5 (18)
O1—C5—C4	108.7 (9)	O2'—C7'—H7'1	110.8
O1—C5—H5A	110.0	C6'—C7'—H7'1	110.8
C4—C5—H5A	110.0	O2'—C7'—H7'2	110.8
O1—C5—H5B	110.0	C6'—C7'—H7'2	110.8
C4—C5—H5B	110.0	H7'1—C7'—H7'2	108.9
H5A—C5—H5B	108.3	O2'—C8'—C9'	110.9 (17)
O1—C6—C7	108.6 (8)	O2'—C8'—H8'1	109.5
O1—C6—H6A	110.0	C9'—C8'—H8'1	109.5
C7—C6—H6A	110.0	O2'—C8'—H8'2	109.5
O1—C6—H6B	110.0	C9'—C8'—H8'2	109.5
C7—C6—H6B	110.0	H8'1—C8'—H8'2	108.0
H6A—C6—H6B	108.4	O3'—C9'—C8'	107.9 (14)
O2—C7—C6	109.5 (9)	O3'—C9'—H9'1	110.1
O2—C7—H7A	109.8	C8'—C9'—H9'1	110.1
C6—C7—H7A	109.8	O3'—C9'—H9'2	110.1
O2—C7—H7B	109.8	C8'—C9'—H9'2	110.1
C6—C7—H7B	109.8	H9'1—C9'—H9'2	108.4
H7A—C7—H7B	108.2	O3'—C10'—C11'	107.8 (15)
O2—C8—C9	109.8 (7)	O3'—C10'—H10C	110.1
O2—C8—H8A	109.7	C11'—C10'—H10C	110.1
C9—C8—H8A	109.7	O3'—C10'—H10D	110.1
O2—C8—H8B	109.7	C11'—C10'—H10D	110.1
C9—C8—H8B	109.7	H10C—C10'—H10D	108.5
H8A—C8—H8B	108.2	O4'—C11'—C10'	110.3 (12)
O3—C9—C8	110.4 (8)	O4'—C11'—H11C	109.6
O3—C9—H9A	109.6	C10'—C11'—H11C	109.6
C8—C9—H9A	109.6	O4'—C11'—H11D	109.6
O3—C9—H9B	109.6	C10'—C11'—H11D	109.6
C8—C9—H9B	109.6	H11C—C11'—H11D	108.1
H9A—C9—H9B	108.1	O4'—C12'—C13'	109.3 (18)
O3—C10—C11	107.8 (8)	O4'—C12'—H12C	109.8
O3—C10—H10A	110.2	C13'—C12'—H12C	109.8
C11—C10—H10A	110.2	O4'—C12'—H12D	109.8
O3—C10—H10B	110.2	C13'—C12'—H12D	109.8
C11—C10—H10B	110.2	H12C—C12'—H12D	108.3
H10A—C10—H10B	108.5	O5'—C13'—C12'	105.6 (15)
O4—C11—C10	109.8 (9)	O5'—C13'—H13C	110.6
O4—C11—H11A	109.7	C12'—C13'—H13C	110.6
C10—C11—H11A	109.7	O5'—C13'—H13D	110.6
O4—C11—H11B	109.7	C12'—C13'—H13D	110.6
C10—C11—H11B	109.7	H13C—C13'—H13D	108.8
H11A—C11—H11B	108.2	O5'—C14'—C15'	112 (2)
O4—C12—C13	107.1 (9)	O5'—C14'—H14C	109.1
O4—C12—H12A	110.3	C15'—C14'—H14C	109.1
C13—C12—H12A	110.3	O5'—C14'—H14D	109.1
O4—C12—H12B	110.3	C15'—C14'—H14D	109.1
C13—C12—H12B	110.3	H14C—C14'—H14D	107.9
H12A—C12—H12B	108.6	O6'—C15'—C14'	106.6 (19)
O5—C13—C12	110.9 (11)	O6'—C15'—H15C	110.4
O5—C13—H13A	109.5	C14'—C15'—H15C	110.4
C12—C13—H13A	109.5	O6'—C15'—H15D	110.4
O5—C13—H13B	109.5	C14'—C15'—H15D	110.4
C12—C13—H13B	109.5	H15C—C15'—H15D	108.6
H13A—C13—H13B	108.0	C1—N1—Zn1	168.0 (4)
O5—C14—C15	108.4 (9)	C2—N2—Zn1	173.6 (6)
O5—C14—H14A	110.0	C3—N3—Zn1	157.0 (7)
C15—C14—H14A	110.0	H4E—N4—H4F	109 (5)
O5—C14—H14B	110.0	H4E—N4—H4G	119 (5)
C15—C14—H14B	110.0	H4F—N4—H4G	107 (5)
H14A—C14—H14B	108.4	H4E—N4—H4H	92 (5)
O6—C15—C14	109.4 (11)	H4F—N4—H4H	120 (5)
O6—C15—H15A	109.8	H4G—N4—H4H	110 (5)
C14—C15—H15A	109.8	S3^i^—S3—C3	73.6 (3)
O6—C15—H15B	109.8	N1—Zn1—N1^i^	115.4 (2)
C14—C15—H15B	109.8	N1—Zn1—N2	107.82 (13)
H15A—C15—H15B	108.2	N1^i^—Zn1—N2	107.82 (13)
C6'—O1'—C5'	112.8 (17)	N1—Zn1—N3	108.70 (13)
C8'—O2'—C7'	106.5 (15)	N1^i^—Zn1—N3	108.70 (13)
C10'—O3'—C9'	111.7 (14)	N2—Zn1—N3	108.2 (2)
C12'—O4'—C11'	114.2 (14)	H1W—O1W—H2W	120 (2)
C14'—O5'—C13'	107.7 (17)	H2W—O1W—H3W	152 (10)
C15'—O6'—C4'	111.2 (18)	H1W—O2W—H4W	152 (10)
O6'—C4'—C5'	108 (2)	H3W—O2W—H4W	121 (2)
			
C15—O6—C4—C5	174.9 (8)	O1'—C6'—C7'—O2'	−68 (3)
C6—O1—C5—C4	173.1 (8)	C7'—O2'—C8'—C9'	−175.8 (18)
O6—C4—C5—O1	61.5 (10)	C10'—O3'—C9'—C8'	−178.1 (15)
C5—O1—C6—C7	174.5 (8)	O2'—C8'—C9'—O3'	64 (2)
C8—O2—C7—C6	−176.9 (8)	C9'—O3'—C10'—C11'	−174.2 (15)
O1—C6—C7—O2	−63.5 (11)	C12'—O4'—C11'—C10'	174.0 (14)
C7—O2—C8—C9	−174.4 (8)	O3'—C10'—C11'—O4'	−62.7 (18)
C10—O3—C9—C8	176.1 (8)	C11'—O4'—C12'—C13'	171.2 (15)
O2—C8—C9—O3	64.5 (10)	C14'—O5'—C13'—C12'	−174.0 (16)
C9—O3—C10—C11	−179.8 (8)	O4'—C12'—C13'—O5'	68 (2)
C12—O4—C11—C10	−178.8 (9)	C13'—O5'—C14'—C15'	−175.8 (19)
O3—C10—C11—O4	−65.3 (10)	C4'—O6'—C15'—C14'	179.3 (18)
C11—O4—C12—C13	179.6 (8)	O5'—C14'—C15'—O6'	−62 (2)
C14—O5—C13—C12	−175.7 (9)	S3^i^—C3—N3—Zn1	67 (3)
O4—C12—C13—O5	67.7 (11)	S3—C3—N3—Zn1	−67 (3)
C13—O5—C14—C15	−179.8 (8)	S3A—C3—N3—Zn1	180.0
C4—O6—C15—C14	−174.1 (8)	N3—C3—S3—S3^i^	156 (3)
O5—C14—C15—O6	−63.7 (11)	S3A—C3—S3—S3^i^	−62.2 (12)
C15'—O6'—C4'—C5'	176.4 (18)	C1—N1—Zn1—N1^i^	170.5 (15)
C6'—O1'—C5'—C4'	175.5 (17)	C1—N1—Zn1—N2	−69.0 (17)
O6'—C4'—C5'—O1'	62 (2)	C1—N1—Zn1—N3	48.1 (17)
C5'—O1'—C6'—C7'	176.4 (18)	C3—N3—Zn1—N1	−116.85 (12)
C8'—O2'—C7'—C6'	−177.5 (18)	C3—N3—Zn1—N1^i^	116.85 (12)

Symmetry code: (i) *x*, −*y*+1/2, *z*.

### Hydrogen-bond geometry (Å, º)

**Table:**

*D*—H···*A*	*D*—H	H···*A*	*D*···*A*	*D*—H···*A*
N4—H4*H*···O1	0.88 (1)	2.44 (4)	3.069 (8)	129 (4)
N4—H4*E*···O2	0.87 (1)	2.06 (1)	2.934 (8)	176 (5)
N4—H4*G*···O4	0.88 (1)	1.97 (2)	2.829 (7)	166 (4)
N4—H4*G*···O5	0.88 (1)	2.53 (4)	3.010 (8)	115 (3)
N4—H4*H*···O6	0.88 (1)	2.05 (3)	2.850 (8)	150 (5)
N4—H4*F*···S1^ii^	0.87 (1)	2.63 (2)	3.441 (4)	156 (4)

Symmetry code: (ii) *x*, *y*, *z*−1.
